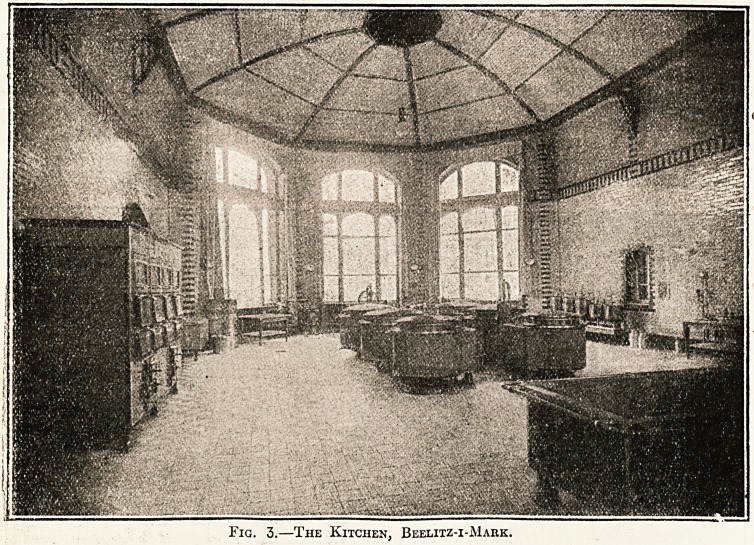# Some Continental Hospitals

**Published:** 1912-04-27

**Authors:** 


### SOME CONTINENTAL HOSPITALS.

### The Rudolf Virchow and Beelitz-i-Mark Kitchens.

There are many special features in the kitchen departments of the great Continental hospitals of the utmost interest to and worthy of the close attention of the English hospital worker. The great thought and attention given to the design, construction, and equipment of the buildings for the treatment of the sick is given equally to those buildings in which the food is prepared and served; and the large sites on which the Continental hospitals are usually erected enable adequate space to be devoted to the buildings for these purposes. As a rule the kitchens are contained in buildings entirely separate and detached from the administrative offices and rooms, and are placed as near as possible to the centre of the hospital, so as to facilitate the service, yet at the same time to be convenient of access for tradespeople. It is thus possible to make the kitchen department complete in itself with all stores and necessary rooms only accessible to the kitchen staff and independent of the other hospital services.

The principle of the food service is the same as in England, the cooked food being conveyed from the main kitchen to the ward kitchens in each pavilion, from where it is served to the patients. In France the kitchen blocks are very often connected to the patients' pavilions and the residential buildings by subways in which tram lines are laid, along which the food is conveyed in waggons to lifts which raise it to the various floors. In Germany, more often, the food service from the kitchen block to the pavilions is conducted through the open, the food contained in water-jacketed vessels being placed in zinc-lined food waggons, which are well insulated against the loss of heat. In the smaller hospitals and clinics the kitchen will very often be contained in an outshoot or in the basement, so that the food service will be conducted either by lifts or along the corridors.

Fig. 1.—Ground-Floor Plan of the Kitchen Block, the Virchow Hospital, Berlin (page 98).

1, Central Kitchen; 2, Roasting Kitchen; 3, Servery; 4, Scullery; 5, Crockery; 6, Pantry; 7, Housekeeper; 8, Milk-room; 9, Meat Preparing; 10, Meat Store; 11, Groceries; 12, Vegetable Scullery; 13, Roasting Utensils; 14, Steward; 15, Office; 16, Staff Dining-room.

Fig. 2.—The Main Kitchen, The Virchow Hospital, Berlin.

### The Kitchen Unit in Berlin.

One of the best kitchen departments on the Continent is found at the Rudolf Virchow Hospital, Berlin, which is in many respects the finest hospital in the world, and in which accommodation is provided for 2,000 patients and a staff numbering upwards of 700. Fig. 1 shows the ground-floor plan of the department, which is contained in a detached block close to the centre of the hospital. In the centre of the block is the main kitchen, 56 ft. long by 44 ft. wide, communicating directly with the servery, to which the food waggons come to convey the food to the pavilions. To one side of the main kitchen, opening from a corridor, is the roasting kitchen, with a room for utensils adjoining, the vegetable scullery, meat-preparing room, meat store, grocery and provision store, and the steward's office, while to the other side is the scullery, crockery store, milk room, two pantries, housekeeper's room, office, and kitchen-staff dining room. In the basement with direct access from outside are the storerooms for vegetables, potatoes, and meat, with refrigerating apparatus; rooms for curing and smoking meat; rooms for the storage of beer, wine, and a complete mineral-water manufactory; numerous other store rooms, and a dining room, with dressing room and bath, for the male kitchen staff. On the first floor are the apartments for the head-cook, cook, and thirty-six kitchen maids, while on the roof floor are the apartments for seventeen male kitchen staff.

Fig. 2, which is a view of the main kitchen looking towards the servery, will give some idea of the size and of the equipment of the department. In this hospital all boiling is done by steam and all roasting by gas. The main kitchen contains twenty-four boiling-pans of various sizes, with a total capacity of 2,100 gallons, for the preparation of soup, meat, vegetables, potatoes, milk, coffee, etc., eight smaller boiling-pans with tilting contrivances in two groups of four, a gas range, and other fittings, while in the front portion of the kitchen are three very large hot-plates, a hot-closet, and the three serving windows. The adjoining roasting and baking kitchen contains two gas ovens with twenty six compartments, a three-compartment potato steamer, a roasting grid, two large *bains-marie*, each with five compartments for the keeping hot of roast and boiled meats, a chopping block, two large hot-plates, and other fittings.

### Sanatorium Kitchen in Germany.

Another fine kitchen is illustrated in fig. 3, which shows the main kitchen for the consumptive sanatoria at Beelitz-i-Mark, belonging to the Berlin Insurance Company. This kitchen, which is 50 ft. long by 35 ft. wide, is contained in a detached block situated in a convenient position between the men's and the women's pavilions, and provides for about 600 persons. In the centre of the floor are seen the eight boiling-pans with a total capacity of 550 gallons; in the foreground is a large range for the cooking of single portions; against one wall is a large coal oven with eight compartments, and further along a *bain-marie*, while against the opposite wall is a group of five tilting-pans of varying capacity.

Fig. 3.—The Kitchen, Beelitz-i-Mark.

### The Cooking Apparatus.

It will thus be seen that the cooking apparatus employed in the Continental hospitals comprises boiling-pans, ovens and ranges for roasting, and apparatus for keeping the cooked food hot until its conveyance to the pavilions. The boiling-pans are usually grouped together in the centre of the kitchen, and so arranged that they can easily be attended to and cleaned. They are steam-jacketed, consisting of an inner and an outer boiler, and so arranged that when necessary, as in the case of milk or fish, a water bath instead of steam may be employed for cooking. The outer boiler is constructed of cast iron well insulated with cork, and covered with an enamelled sheet-iron casing. The inner boiler is usually nickel-plated, but in the case of vegetable boilers it is often of tin-plated copper, and in the case of milk or coffee boilers of smooth cast iron. The lids are self-balancing, constructed of aluminium plate and fitted with vapour extracts to the common condenser and safety valves. Draw-off cocks are provided to each boiler, and between each two is a standard with hot and cold water supply. All pipes are brought up from below the floor, a proper basement being provided for the piping. Ranges and ovens for roasting are either heated by gas or fuel, gas perhaps being the more usual. The apparatus for keeping the cooked food hot comprises *bains maries*, which are usually constructed of copper, the water bath being heated by steam coils, and vessels being provided for milk, beef-tea, coffee, etc., and portions of meat. The hot plates and closets are heated by steam.

In the various preparation rooms and sculleries one finds that labour-saving machines are greatly used, such as machines for the washing and pealing of potatoes, the preparation of vegetables, coffee mills, mincing machines, sausage-stuffing machines, and plate-washing and knife-cleaning machines, all whenever possible being electrically driven. Wash-up sinks in sculleries are usually constructed of durana metal, while those in vegetable sculleries or reservoirs for live fish are constructed of marble slabs.

From the photographs the cleanliness and lightness of the kitchens will be perceived. All floors are tiled, and the walls are faced either with white glazed bricks or else have white glazed tile dadoes. Heating is as a rule by steam radiators, special provision for ventilation and the extraction of steam is always made, and the artificial lighting is almost invariably by electricity.

## Figures and Tables

**Fig. 1. f1:**
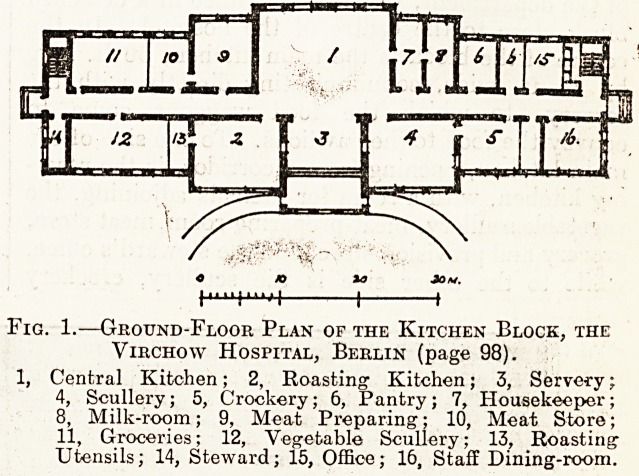


**Fig. 2. f2:**
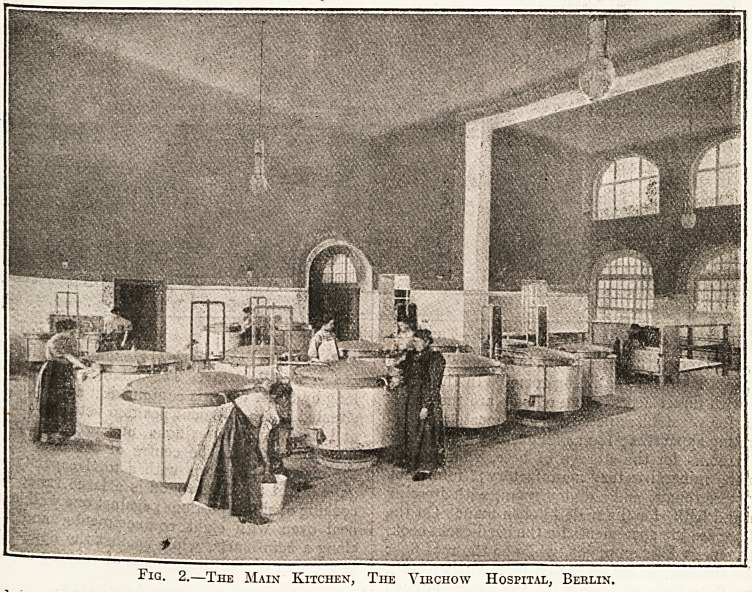


**Fig. 3. f3:**